# Building consensus about eHealth in Slovene primary health care: Delphi study

**DOI:** 10.1186/1472-6947-11-25

**Published:** 2011-04-18

**Authors:** Rade J Iljaž, Matic Meglič, Igor Švab

**Affiliations:** 1 Department of Family medicine, Medical Faculty, University of Ljubljana, Poljanski nasip, 58 Ljubljana, Republic of Slovenia; 2 Institute of Public Health of the Republic of Slovenia, Trubarjeva 2, Ljubljana, Republic of Slovenia

## Abstract

**Background:**

Slovenia's national eHealth strategy aims to develop an efficient, flexible and modern health care informatics framework that would be comparable to the most successful EU countries. To achieve this goal, the gap between availability and usage of information and communication technology by primary care physicians needs to be reduced.

As recent efforts show, consensus on information and communication technology purpose and usage in primary care needs to be established before any national information and communication technology solutions are developed.

The aim of this study was to identify the most appropriate measures in implementation of Slovene national eHealth strategy and to suggest an appropriate model for success by using the three round Delphi study.

**Methods:**

An e-mail based, three-round Delphi study was undertaken to achieve consensus from a selected sample of nationally recognized experts from the fields of primary health care and medical informatics. The aim of this study was to identify the most appropriate measures and key obstacles in implementation of eHealth in Slovene primary health care by using the Delphi study.

**Results:**

High levels of consensus on the majority of suggested measures were achieved among all study participants, as well as between the subgroups of experts from primary health care and medical informatics. All aims of the three-round Delphi study on eHealth implementation in Slovenian primary health care were achieved.

**Conclusions:**

The three round decision Delphi process has proven to be effective for developing outcomes, ranking key priorities in primary care eHealth development, and achieving consensus among the most influential experts in that field. This consensus is an important contribution to future national eHealth strategies in the field of primary health care.

## Background

The importance of eHealth services development and their successful implementation in health care has been well known for a long time. In all developed health care systems agreement has been reached about the importance of incentives for new information and communication technology usage, particularly in ambulatory health care [1-4]. Nevertheless, there is often a significant discrepancy between policy and practice [5-9]. Healthcare professionals play a key role in successful acceptance, implementation in everyday practice, and continuous development of various eHealth services and applications [4,6,10-16].

At the end of 2007 the majority of primary health care (PHC) practices in the EU used computers and almost 70% of all practices had internet connection. There was a trend of larger practices being better equipped with computers than smaller ones (93% versus 84%). Denmark, the Netherlands, Finland, Sweden and the UK have also established themselves as the European front-runners in eHealth penetration in PHC practice. The average discrepancy between availability and use of computers in the patient examination room was 12%, ranging from 0% in Finland where all GPs use computers in writing reports and orders, to 54% of practices in Slovenia, which has the biggest gap in the EU [7]. Taking into account other eHealth development parameters (use of broadband internet, practice website, electronic storage and transfer of administrative patient data, electronic connection to other health actors etc.), Slovenia is however comparable to many other EU countries [7].

### Slovenia's primary health care and eHealth strategy

In Slovenia, the majority of primary health care is delivered and coordinated by local public Primary Health Care Centres (PHCCs). About a third is delivered by independent contractors to the Health Insurance Institute of Slovenia.

First ambulatory electronic health records were introduced in Slovenia in the early 1990s and were designed primarily for administrative purposes. Consequently, primary care physicians' acceptance of contemporary information and communication technology (ICT) and health information systems (HIS) remained low throughout the next decade [17-19].

Slovenia's eHealth strategy was introduced in 2005 to develop an efficient, flexible and modern national health care informatics system, comparable to the most successful EU countries [20,21]. The strategy and its operational plan had three main focuses: (1) to upgrade the basic information infrastructure for safe and transparent exchange of information between patients, health care service providers and payers; (2) to define and introduce interoperable electronic health care records and integrate them into daily work of medical and allied professionals with patients, and (3) to introduce and sustain the national health care portal and implement data exchange between patients, various healthcare providers, payers and others.

In achieving this goal, the role of primary care physicians was very important. The problem of implementation of ICT in primary care is that despite best research evidence, the implementation is often not successful because the proposals are not accepted [22,23]. Because of that, there is a need to find consensus between different players in this activity.

### Goal and objectives

The aim of this study was to identify the most appropriate measures in implementation of Slovene national eHealth strategy, to identify key obstacles to implementation in PHC, and to suggest an appropriate model for success.

The following hypotheses were made: (1) it is possible to define and rank priorities for eHealth development in PHC by using the Delphi method; (2) there are significant differences between primary health care experts' and medical informatics specialists' ranking of eHealth priorities in Slovenia.

The main objective of the study was to develop a novel method for identifying and ranking the most important and the most feasible eHealth measures in PHC settings by using the three round Delphi study.

## Methods

An e-mail based, three-round decision Delphi study was performed to generate responses and achieve consensus in a selected sample of nationally recognized eHealth experts. Informed consent was obtained from each participant and approval was obtained from Slovene National Ethical Committee.

The Delphi survey was developed in the 1950s. It is a structured and multistage process where a panel of experts are invited to take part in a series of rounds to identify, clarify, and finally achieve consensus on a particular issue. Each subsequent set of non-leading, unambiguous statements is built on the responses to the preceding ones. Consensus is sought through the feedback of information and iteration, and the process is terminated when consensus is reached.

Anonymity offered by Delphi can reduce the inhibition normally occurring in decision-making as individuals are more open with their answers [24].

There were two main reasons to choose Delphi method as our research tool. Firstly, the Delphi technique has been found effective in the past in raising and measuring group consensus about medical information technology usage within health care [25-29]. Secondly, the outcome in the decision Delphi is focused on decision making in fields that are strongly susceptible to change and where one or more of the following occurs: (1) there is more influence by individual decision makers than by underlying rules; (2) the field of interest is relatively new or is driven by new developments; or (3) the scientific field of interest is small and relatively self-contained. The panel should include a high percentage of decision makers in the considered field [30,31].

In primary healthcare as well as in eHealth it is often the method of choice for developing consensus on national level [10,32-37].

### Organization of the study

The monitoring of the study was performed by a steering committee that identified the experts to be invited to participate in the Delphi process and that also oversaw the project.

The project group, consisting of two experts from PHC and two medical informatics (MI) specialists, prepared and analyzed the questionnaires. Communication with participants was performed via e-mail with anonymity of written answers assured.

### Sample

The sample included two groups of experts. The selection criteria were:

1) At least 10 years of professional work in primary health care or 5 years in medical informatics,

2) High position in a professional hierarchy (professional association or institution), and/or

3) Adequate knowledge of the organization of Slovenian PHC.

The estimate of sample size was based on literature review and reports from similar studies [10,31-33,35,36,38,39]. We anticipated approximately an 80% response rate from 13 invited experts in the both fields - of PHC and medical informatics - this would ensure the required significance.

PHC experts were recommended by the Department of Family Medicine, Medical Faculty, University of Ljubljana. Medical informatics specialists were recommended by members of the Healthcare Informatics Council, an advisory board to the Slovene Ministry of Health. All recommendations were approved by the steering committee, that checked their references.

### Delphi questionnaire development

The first-round questionnaire consisted of semi-structured questions. They were generated based on a literature review performed by a group of experts and from the input of seven focus groups. These focus groups included: three PHC physician groups, two nurse groups and two patient groups.

Questions were grouped into three main themes:

1) *Availability and use of contemporary information technology in family medicine practice*, with next sub-themes: "an expected level of computerization in PHC practices" and "the role of state institutions".

2) *Expectations from contemporary ICT*, with sub-themes: "quality of health care services", "health care costs", "satisfaction of medical staff and users of health care services", "paperless healthcare" and "data management and safety of medical data".

3) *Computer supported decision-making, electronic communication with patients and between health care levels *with sub-themes: "e-communication between patient and health care provider", "e-communication among different health care providers" and "computer supported decision-making".

The draft questionnaire was given to a group of four experts for comments (two from PHC and two from medical informatics). They were asked to rate the questions in two dimensions, clarity and importance, and to comment on the questions. The questions acceptable to all experts were included in the first questionnaire.

The questions were in the form of statements and levels of agreement with the statements were assessed on a 1 to 9 Likert scale.

### The Delphi consensus process

Steering committee planned at least three Delphi rounds. Additional rounds were to be performed in case of less than 80% agreement on all items after round three. Agreement for single items was defined by: (1) median value of six or higher, (2) inter-quartile rank (IR) of three or less, and (3) no statically significant differences between MI and PHC subgroup of experts.

### First round

The main task of all invited experts was to assess the *clarity *and *importance *of each question. This included comments about the questions, including particular terminology and vocabulary related to ICT, medical and administrative terms.

The first round of questionnaires was analyzed using simple statistics: mean, standard deviation (SD), and rank. After this analysis, the questionnaire was modified according to the following criteria.

The exclusion criteria for a statement were: (1) median - all expert ranks lower than five; or (2) IR - higher than five; or (3) median - lower than six, with IR equal or higher than four.

The criteria for modification were: (1) median - six or seven, with IR from three to five with relevant written arguments from the panellists; (2) same meaning of the question; (3) consensus of all strategic project group members; and (4) no limitation on the number of modified questions.

The criteria for adding new questions after the first round were the following: (1) only questions developed by first round participants, which had been reviewed and confirmed by the strategic project group; and (2) no more than 15% of the total number of first-round questions.

Criteria for previously defined exclusion, modification and addition applied to both assessment areas, namely clarity and importance of each statement.

### Second round

The second round questionnaires were also sent by e-mail. The experts were asked to assess the *importance *and *feasibility *of each suggested measure and to complete a short demographic survey. Levels of agreement were again assessed on a 1 to 9 Likert scale. No feedback was provided concerning the results of the first round. The experts that didn't return the questionnaire were reminded by phone after 10 days. The responses were analyzed in order to develop the third questionnaire (except for newly generated questions), based on interquartile range (IR), median, and mean. The criteria for the exclusion or modification of a particular question after the second round were the same as in the first round. In addition, the number of modified questions was limited to 10.

The criteria for the addition of new questions after the second round were the following: (1) relevant arguments by second-round panelists on a particular topic; (2) consensus of all strategic project group members; and (3) limited to 10% of all second-round questions.

### Third round

In the third round, the participants were asked to review their responses from the previous round and to contrast them using consensus results of the group. The questionnaire was also accompanied with statistical data and comments from the second round. The following information was included: (1) median; (2) consensus; and (3) IR. The respondents' initial ratings in the second round questionnaire were highlighted. They could then change their initial rating. The participants whose score on any statement was outside the group consensus were asked to briefly explain the reasons for their position.

### Statistical analysis

Two methods were used to assess consensus building. A rating-scale was used to indirectly measure agreement (based on IR) and inter-round comparison of results. Comparison of subgroup results for each question was also performed using non-parametric statistical technique (Wilcoxon rank sum test).

After the last round, descriptive statistics was used to analyze the final results [40,41].

In the second step differences between subgroups of PHC professionals and medical informatics experts were analysed using the Wilcoxon rank sum test.

The third step of the analysis involved Cronbach's alpha analysis to determine the internal consistency of answers in both rounds. Finally, the Wilcoxon signed-rank sum test was used to determine the overall success of the Delphi process. Statistical differences on the importance and feasibility of all rated statements in the second and third round were analyzed.

All quantitative data analyses were performed using Data Analysis Plus 3.0 Statistical Software ad-ins for Microsoft Excel [42].

After the descriptive statistical analysis of answers to third round statements, they were ranked by order according to median, IR, SD and mean. The classification was made on both rating dimensions, namely importance and feasibility.

## Results

Thirteen PHC professionals and an equal number of medical informatics experts were invited to participate in the survey. Twenty-four of those participated in first round: 13 were PHC physicians and 11 were key stakeholders in the field of Slovenian medical informatics. Twenty-two experts participated in each of next two rounds: 12 were PHC physicians and 10 were medical informatics (MI) experts. We achieved 92.31% response rate in first round and 84.61% response rate in second and third round.

The average age of the MI experts was 47.7 years and average professional experience in the MI field was 19.4 years. The average age of the PHC experts was 46 years and average professional experience in primary care was 20 years. The percentage of non-answered or incompletely answered questions was 3.4% in the first round, 4.5% in the second round and 2.2% in the third round.

### First round

Twenty-four experts reviewed and completed the first-round questionnaire, which have sorted 96 questions into three groups. After the first round, 13 questions (13.5%) were modified, 8 questions (8.3%) were excluded and 17 new questions (17.7%) were added according to the above methodological criteria.

### Second round

This consisted of 107 questions, divided into three subgroups. In the second and third round, 22 of 24 first-round's panellists assessed the feasibility and importance of suggested measures. After the second round, 12 additional questions were excluded (11.2%), 7 questions modified (6.54%), and 10 new questions added (9.3%). At the end, the third round questionnaire contained 105 questions, of which 95 were the same or slightly modified from the second round.

The comments concerning second round questions that strongly influenced the modification and addition of new statements were the following:

- 'I strongly believe eHealth should enable PHC nurses to take over more medical and less administrative duties.' - PHC physician

- 'Awareness of top management and politicians is crucial for any ICT success.' - MI expert.

- 'We need different methods to stimulate physicians who actively use electronic health records (EHR) and to reduce the number of physicians using paper.' - MI expert

- 'What about national ICT standards for data exchange, software, certification of ambulatory EHRs, etc.?' -MI expert.

- 'Without a national health information system, including an uniform health network, full EHR implementation is impossible.' - MI expert

- 'The creation of a national coordinating team for ICT implementation in primary health care is necessary.' - PHC physician.

- 'Why do existing ambulatory EHRs still not allow for the structured entry of medical data?' - PHC physician.

### Third round

In the third round, all of 22 second round's panellists again assessed the feasibility and importance of the statements. The third round measures from both ranking fields (importance and feasibility), which ranked the best and the worst, are given in Tables 1, 2, 3 and 4.

**Table 1:** The 10 best-ranked measures based on importance

Rank	Suggested measure	Median	IR	SD
1	All PHC practices should be equipped with at least two adequately efficient personal computers.	9	0	0.21
2	A general practitioner should make records about every patient's *vaccinations *in the national electronic medical health record.	9	0	0.29
3	A general practitioner should make records about every patient's *allergies *in a visible section of the ambulatory electronic record.	9	0	0.43
4	A general practitioner should make records about every patient's body weight or body mass index (BMI) in a visible section of the ambulatory electronic record, at least once every 5 years.	9	0	0.50
4	A general practitioner should make records about every patient's *vaccinations *in the ambulatory health electronic record.	9	0	0.50
4	All PHC practices should use certified computer decision-support programs for decision-making, therapy, prescribing and controlling.	9	0	0.50
7	In all PHC practices, doctors should write therapeutic instructions, referrals and the most important clinical findings during examination into ambulatory electronic records.	9	0	0.61
8	All PHC practices should have broadband access to the worldwide web.	9	0	0.64
9	The Ministry of Health and health insurance institutions should methodically educate and inform health care providers about benefits of modern informational technologies.	9	0	0.77
10	In all PHC practices, nurses should write administrative data during the consultation into ambulatory electronic records.	9	0	0.87

**Table 2:** The 10 worst-ranked measures, based on importance

Rank	Suggested measure	Median	IR	SD
105	Computer decision-support programs for family doctors should be adopted and verified by the Slovenian Medical Informatics Association.	5	3	2.19
104	The usage of national electronic medical records in all practices of PHC would reduce expenses of commercial insurance companies in the Republic of Slovenia.	5.5	3.5	2.03
103	The usage of improved ambulatory electronic records in all PHC practices would reduce expenses of commercial insurance companies in the Republic of Slovenia.	6	4	2.05
102	Clinical specialists should ensure (for a fee) e-consultation to all family physicians in their region.	6	3.25	2.17
101	Usage of national electronic medical records in all practices of PHC would reduce operating costs of The Health Insurance Institute of Slovenia.	6	3	1.91
100	The use of national electronic medical records in all PHC practices would reduce operating costs of The Health Insurance Institute of Slovenia.	6	2.5	2.13
99	Supervision of secure and confidential data handling in national electronic medical records should be carried out by a special entity at The Slovenian Ministry of Health.	6	2.25	2.11
98	All PHC and clinical departments should send relevant medical documentation to each other by local area network (LAN).	6	2	1.73
97	Usage of improved ambulatory electronic records in all practices of primary health care would significantly reduce health care costs in the Republic of Slovenia.	6	1	1.51
96	A general practitioner should record every patient's illicit drug usage in the national electronic medical record.	7	6.25	2.96

**Table 3:** The 10 best-ranked measures, based on feasibility

Rank	Suggested measure	Median	IR	SD
1	All PHC practices should be equipped with at least two adequately efficient personal computers.	9	1	0.67
2	In all PHC practices nurses during the consultation should write administrative data into ambulatory electronic records.	9	1	0.86
3	Computer decision-support programs for family doctors should be adopted and verified by the Extended Professional Board of Slovene Family Physicians.	9	1	0.89
4	All PHC practices should have broadband access to the worldwide web.	9	1	1.37
5	In all PHC practices nurses should write some medical data into the *ambulatory electronic health record *during the consultation.	9	1.25	1.25
6	A general practitioner should make records about every patient's *allergies *in a visible section of the ambulatory health electronic record.	9	2	1.41
7	A general practitioner should make records about every patient's body weight or body mass index (BMI) in a visible section of the *ambulatory health electronic record*, at least once every 5 years.	9	2	1.66
8	A general practitioner should make records about every patient's vaccinations in the *ambulatory electronic health record.*	8.5	2	1.15
9	All PHC practices should receive cost coverage from The Slovenian Ministry of Health or The Health Insurance Institute of Slovenia for the purchase of computer equipment.	8.5	3.75	2.31
10	Regarding the most common medical conditions, a family practitioner during the consultation should be able to use structured order-data entry modules in the *ambulatory electronic health record*.	8	2	1.36

**Table 4:** The 10 worst-ranked measures, based on feasibility

Rank	Suggested measure	Median	IR	SD
105	Usage of improved ambulatory electronic medical records in all PHC practices would reduce expenses of commercial insurance companies in The Republic of Slovenia.	5	3.5	2.33
104	The general practitioner should record every patient's illicit drug misuse in the national electronic medical record.	6	6.25	2.96
103	The Slovenian Ministry of Health should offer all PHC practices standardized computer software for aid in professional decision-making, *free of charge*.	6	4.25	2.51
102	Usage of national electronic medical records in the PHC practices would be accompanied by an increased satisfaction of health care users regarding health care services in primary health care.	6	3.5	1.85
101	Clinical practices should offer some tele-medical services to their patients according to well-defined rules and widely-accepted standards.	6	3.5	1.91
100	The Chamber of Health and other medical associations should actively participate in lowering costs in the purchase and maintenance of ambulatory medical records in general/family medicine practices.	6	3	2.38
99	Usage of improved ambulatory electronic records in all PHC practices would significantly reduce health care costs in The Republic of Slovenia.	6	3	2.11
98	*All *data corresponding to the patient from national electronic medical records should be freely accessible to other health care providers when in contact with the patient.	6	3	2.06
97	Usage of national electronic medical records in all PHC practices would reduce operating costs for an individual family practice.	6	3	1.84
96	Usage of national electronic medical records in all PHC practices would reduce operating costs of The Health Insurance Institute of Slovenia.	6	2.5	2.13

Large values of IR mean that the first and third quartiles are far apart, indicating high variability of the answers and consequently, indicating a high level of disagreement among panellists.

### Comparisons (Tables 5 and 6)

**Table 5:** Suggested measures, based on importance, with significant statistical differences in ranking between subgroups of PC and MI experts

Rank of measure's importance	Rank of measure's feasibility	Suggested measure	Medi-an	IR rang	P
**37**	35-80	In all PHC practices doctors should write administrative data into ambulatory electronic health records during the consultation.	9	2	< 0.03
**45-80**	9	All PHC practices should receive cost coverage from The Slovenian Ministry of Health or The Health Insurance Institute of Slovenia for the purchase of computer equipment.	8	5.5	< 0.04
**45-80**	35-80	All PHC practices should receive cost coverage from The Ministry of Health or The Health Insurance Institute of Slovenia for the purchase of computer software for ambulatory electronic records.	8	5	< 0.03
**45-80**	35-80	All PC practices should receive cost coverage from The Ministry of Health or The Health Insurance Institute of Slovenia for the purchase, maintenance and updates of computer software for ambulatory electronic records.	8	5	<0.04
**40-44**	35-80	All PHC practices should be offered at a reasonable price (by The Ministry of Health) standardized computer software for aid in professional decision-making.	8.5	1.25	<0.03
**45-80**	35-80	Usage of national electronic health records in PHC practices would enable accurate monitoring and rewarding of health care quality.	8	2.25	<0.02
**40-44**	30-34	The majority of data in ambulatory electronic records should be accessible free of charge to the health care users to whom the data corresponds.	8.5	3	<0.05
**45-80**	101	Clinical practices should make accessible to their patients some tele-medical services, according to well-defined rules and widely-accepted standards.	8	1	<0.01

**Table 6:** Suggested measures, based on feasibility, with significant statistical differences in ranking between subgroups of PC and MI experts

Rank of measure's importance	Rank of measure's feasibility	Suggested measure	Medi-an	IR rang	P
**93**	84-95	Usage of ambulatory electronic health records in the practices of primary health care would be accompanied by increased satisfaction of health care users regarding health care services in primary health care.	6	2.5	<0.04
**100**	84-95	Usage of national electronic medical records in the practices of primary health care would be accompanied by increased satisfaction of health care users regarding health care services in primary health care.	6	3.5	<0.04
**35-80**	45-80	All general/family medicine practices should make available patient appointments by e-mail.	7	2	<0.05

Ninety-five statements assessed in the last two rounds were included, rating both the importance and feasibility of suggested measures. Significant statistical differences were found between two subgroups concerning eight measures based on importance and three measures based on feasibility (Tables 5 and 6).

Finally, the signed-rank sum test was used to compare: (1) medians for the importance of all the unchanged statements (n = 95) in the second and third round questionnaires; (2) medians for the feasibility of all the unchanged statements (n = 95) in the second and third round questionnaires; (3) IR for the importance of all the unchanged statements (n = 95) in the second and third round questionnaires; and (4) IR for the feasibility of all the unchanged statements (n = 95) in the second and third round questionnaires. Statistically significant differences (p < .001) were found in all of these four analyses.

## Discussion

### Discussion on methodology

The three round Delphi process proved to be effective in developing outcomes and indicators that were then used in the definition of eHealth content and ranking key priorities in PC eHealth development. High response rates of core PHC and MI experts who all participated in last two rounds helped achieving the consensus. We confirmed both hypotheses postulated at the beginning of the study.

The methodological approach adopted from previously published Delphi studies proved adequate in testing the hypotheses, as well as in achieving the main goals of the study. This methodology was successful at some critical stages of the study - such as achieving: (1) an adequate response rate and - consequently - a sufficient number of participants in all three rounds; (2) a balanced number of experts from the fields of PHC and medical informatics; (3) a low percentage of unanswered or incompletely answered questions, an important condition for the assessment of the questionnaires after each round, as well for the final statistical analysis; and (4) a sufficient number of questions reflecting the major information-technology priorities of Slovene primary health care.

Responses to only 1 of the first 15 statements ranked by feasibility and importance were found to be significantly different between the PHC and MI experts. From this we can conclude that the consensus assessment methods used were complementary. Given the large number of statements in the study, the optimal possible assessment methods were presumably chosen.

The numerous written comments by the experts, particularly after the second round, helped to recognize faster and understand better the relevant eHealth problems and dilemmas of MI and PHC professionals.

Some of the questions in the questionnaire that we have generated were related to the legislative provisions regarding ICT in Slovenia and the answers may have reflected the personal opinions of the respondents regarding Slovenian ICT policy and its acceptance. In our opinion, this has not influenced our goal of building consensus, which was the focus of our study.

### Discussion of ranking and experts' priorities

Very high response rate and small percentage of non-answered or incompletely answered questions as basic requirements for unbiased analysis were achieved.

Participants rated importance significantly higher than feasibility of suggested measures in the second as well third Delphi rounds (p < 0.001). In the third round only seven statements gained the highest median value (9) for feasibility, compared to 39 highest median values (37.1% of the total) for importance. The experts concurred with the majority of measures from the first group ('Availability and usage of contemporary informational technology in family medicine practice'), with the highest median value of all for importance - 61% for these 16 questions. Significantly, only one in 11 suggested measures from the subgroup named 'An expected level of computerization in PHC practices' got a median score of 8.5, while all the others got the median score of 9. All values of the IRs in this subgroup were very low, indicating high level of consensus that this subgroup of measures should be a top priority for ICT development in Slovene PHC.

'Structured data entry' and 'recording of the most important personal health data and conditions in the EHR were two other fields that gained the highest support and consensus of the experts in terms of importance and feasibility. Measures like 'recording patient data about body weight' and 'allergies and vaccination recording' in both - national and local ambulatory electronic health records got the highest support with minimal disagreement (IR < 1).

The third subgroup of measures that experts rated highly for importance was 'Computer supported decision-making,' which scored 6 of the highest median values (66.6%). Experts had little doubt about the importance of computer decision-making support for the most frequent and demanding illnesses, prescriptions, therapy control, and health care prevention. They were much more sceptical about the feasibility of these suggested measures - all these median values were 7 or less.

The experts were unanimous in their strong conviction (IR = 0; Median = 9) that the Supervisory board of family medicine doctors should be the sole professional entity empowered to verify and supervise all software designed for decision-making support in PC. Consequently, they rejected the other four solutions suggested for verification and supervision offered in the second and third Delphi rounds like the Medical Chamber, the National Health Insurance Service, and the Institute for Medical Informatics and the Slovene Medical Informatics Society.

By a wide margin the lowest support from the experts was reserved for subgroup eight measures, under the category 'health care costs'. For importance as well as feasibility this subgroup had an average median value of only 6, and an average IR value of 3. Experts seriously doubted that a wider use of local ambulatory and/or national electronic health records would reduce health care costs.

### Discussion about the implication of consensus findings

Surely, all measures which got sufficient experts' support in both assessed areas need to be implemented in practice. However, it is expected that measures with median 8 or more and IR 2 or less become top eHealth priorities for health authorities.

Some of these measures, like broadband connection and two efficient computers per practice are already reality in almost 90% of Slovene family practices. The others, like recording of very important health data in electronic health records are cheap and unpretentious and consequently have a great implementation potential.

Some of very high ranked measures that could cause substantial changes in workflow of common family practices are to be implemented with care. Such measures are for example physician's and nurse's input of medical data in the EHR.

### Discussion about assessing consensus progress

Significant progress in building consensus in both survey fields - feasibility and importance - has been made during the second and third rounds. The average median value for importance increased from 7.82 to 8.05 and the average median value for feasibility increased from 6.78 to 7.16. At the same time, average IR values decreased from 2.25 to 1.75 and from 2.5 to 2.0 respectively. The statistical significance of higher medians values and lower IR values in the third round was confirmed by the use of non-parametric tests.

Eight questions concerning importance showed statistical differences between the subgroups of PHC physicians and ICT experts. Four of these questions were about the suggested role of Slovene health authorities in the cost policy of ICT use in PHC.

There were statistical differences between the subgroups with respect to increased satisfaction of the PC staff after the full implementation of EHRs. These indicated significantly different expectations about the benefits of IT implementation.

In regard to the goals of the study we suggest that measures that received low support from both subgroups of experts in third round (5.23% of all) should be either excluded or revised significantly. Measures with significantly less support in only one subgroup (10.5% of all) need additional discussion and justifications by the experts that disagreed. The high level of agreement of both subgroups of experts on the feasibility and relevancy of 84.2% measures is expected to make implementation of eHealth much more effective in Slovene PHC. These results indicate that most respondents were interested in the opinions of the group, as well as in moving closer to the perceived consensus.

Cronbach's alpha values for the second round were 0.915 for importance and 0.965 for feasibility. In the third round, Cronbach's alpha values were 0.952 and 0.85 respectively. Such high Cronbach's alpha values indicated a very high level of the internal consistency of answers and reliability of presented items in both rounds.

## Conclusions

This study proves that Delphi process is an efficient way to attain a high level of consensus between health care professionals and experts who are engaged in medical informatics. It also shows that the majority of discrepancies between the needs and priorities of PHC doctors written in the national eHealth strategy could be successfully resolved by the Delphi process. Even though the participating experts came from one country, this shouldn't limit the applicability of study results to Slovenia only due to high similarity of PHC in Slovenia and other EU Member States.

Thus, the results of this study might also provide a basis for eHealth implementation in general, as well as for some more specific interventions.

## Abbreviations

BMI: body mass index; HER: electronic health record; EU: European Union; GP: general practitioner; HIS: health information systems; ICT: information and communication technologies; IR: interquartile rang; LAN: local area network; MI: medical informatics; PHC: primary health care; PHCC: primary health care centre

## Competing interests

The authors declare that they have no competing interests.

## Authors' contributions

RI: Study concept and design, collection of data, statistical analysis and interpretation of data, drafting of the manuscript and critical revision of the manuscript and acquisition of funding. MM: Study concept, design and coordination, collection and interpretation of data, help with drafting the manuscript, critical revision of the manuscript. IŠ: Study concept and design, interpretation of data, critical revision of the manuscript, study supervision.

## Pre-publication history

The pre-publication history for this paper can be accessed here:

http://www.biomedcentral.com/1472-6947/11/25/prepub
